# ArrayPlex: distributed, interactive and programmatic access to genome sequence, annotation, ontology, and analytical toolsets

**DOI:** 10.1186/gb-2008-9-11-r159

**Published:** 2008-11-12

**Authors:** Patrick J Killion, Vishwanath R Iyer

**Affiliations:** 1 Center for Systems and Synthetic Biology, Institute for Cellular and Molecular Biology, Section of Molecular Genetics and Microbiology, University of Texas at Austin, 1 University Station A4800, Austin, Texas 78712, USA

## Abstract

ArrayPlex is a software package that centrally provides a large number of flexible toolsets useful for functional genomics, including microarray data storage, quality assessments, data visualization, gene annotation retrieval, statistical tests, genomic sequence retrieval and motif analysis. It uses a client-server architecture based on open source components, provides graphical, command-line, and programmatic access to all needed resources, and is extensible by virtue of a documented application programming interface. ArrayPlex is available at .

## Abstract 2

ArrayPlex is a software package that centrally provides a large number of flexible toolsets useful for functional genomics.

## Rationale

Although centralized storage of microarray data is provided by a number of databases, such as ArrayExpress, Gene Expression Omnibus, Stanford Microarray Database/Longhorn Array Database, Bioarray Software Environment, and TM4 [1-6], many common downstream analysis procedures remain challenging, especially when reference to large-scale data in external databases is required. Data analysis typically involves association of gene names with systematic and custom annotations, gene ontology information, and genomic DNA sequence, followed by a battery of analyses such as enrichment of functional annotations in gene sets, statistical tests for significance, analysis of *cis*-regulatory motifs and regulator-target relationships. Resources for these tasks are difficult to manually assemble while ensuring they remain error free. Amplifying the challenge is the fact that such analyses are not executed just once, but usually consist of a series of iterations with changing parameters. In order to reduce inefficiency and minimize errors, new algorithms for newly devised data analyses must ideally interface with pre-existing code and algorithms that already satisfactorily address other domains of data analysis.

In an attempt to address this pervasive set of challenges in functional genomics analysis, we developed ArrayPlex, a network-centric software environment chartered with the goal of streamlining the acquisition and up-to-date maintenance of these resources and the ease by which they can be associated with primary microarray data. We illustrate the functionality of ArrayPlex by marshalling systematic annotations and complete genomic sequence information for three organisms: *Homo sapiens*, *Mus musculus*, and *Saccharomyces cerevisiae*. In addition, we have assembled access to a suite of commonly utilized DNA sequence analysis toolsets. ArrayPlex interfaces with all of these bundled resources to provide microarray quality assessments, data visualization, gene annotation retrieval, statistical tests, genomic sequence retrieval and motif analysis. Complete lists of managed resources and toolsets are provided in Tables 1 and 2, respectively.

**Table 1:** Managed resources

Resource name	Source	Related organism
Gene Ontology Descriptors	GO Consortium	Hs, Mm, Sc
Genome sequence	UCSC	Hs, Mm
Hs Gene Ontology assignments	EBI	Hs
Mm Gene Ontology assignments	EBI	Mm
Sc annotations	SGD	Sc
Sc genome sequence	SGD	Sc
Sc Gene Ontology assignments	SGD	Sc

Genomic resources downloaded by the ArrayPlex installation program. Each of these resources is kept up-to-date and is accessible by the ArrayPlex client, command-line modules, and programmatic API. EBI, European Bioinformatics Institute; SGD, Stanford Genome Database; UCSC, University of California, Santa Cruz. Hs, *Homo sapiens*; Mm, *Mus musculus*; Sc, *Saccharomyces cerevisiae*.

**Table 2:** Integrated toolsets

Tool name	Purpose	Download	Reference
AlignAce	Sequence discovery	Acquire	[15]
Avid	Sequence alignment	Acquire	[13]
BLAST	Genomic sequence matching	Bundle	[17]
ClustalW	Sequence alignment	Bundle	[16]
cluster	Hierarchical clustering	Acquire	[18]
MDSCAN	Sequence discovery	Bundle	[20]
MEME	Sequence discovery	Bundle	[19]
fastacmd	Sequence retrieval	Bundle	[17]
rVista	Sequence alignment	Acquire	[14]

The toolsets integrated into the ArrayPlex server environment. The download code of 'Bundle' indicates that the ArrayPlex installation program is capable of downloading the source-code and building the tool during the installation process with no further interaction needed. Alternatively, a code of 'Acquire' indicates that a license agreement is required for download and, thus, the installer of the ArrayPlex server must manually download a file and place it in the proper place on the ArrayPlex server. Documentation is provided for how to acquire and install all toolsets with this requirement.

Our goal was to develop an open-source, robust, and easy to maintain network-centric system that enables the construction of reusable pipelines of complex data analysis procedures. We designed the system to communicate on three levels of interaction: a graphical user interface for interactive data manipulation, a set of command-line analytical modules for script-driven analysis, and a documented Java-based programmatic application programming interface (API). Below we describe the systematic architecture of the ArrayPlex environment and the genomic resources included within it. Additionally, we demonstrate how ArrayPlex has been indispensable in the large-scale analysis of a transcriptional regulatory network.

## System architecture

### Core technology, design, network operation

ArrayPlex was implemented with exclusively open-source technologies. Components were selected to enable creation of an encapsulated system; virtually all of the open source distributable software components required for function are bundled within the installation package.

**Figure 1:**
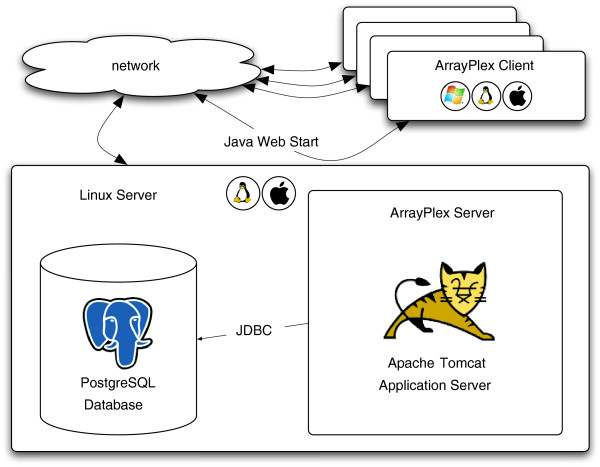
Core technology, high-level overview. The ArrayPlex server is a nearly encapsulated system composed of an embedded Java Runtime Environment and Apache Tomcat application server. The ArrayPlex server requires one external resource, a PostgreSQL relational database server. The ArrayPlex server and PostgreSQL database need not operate on the same computer. The ArrayPlex server operates within the Linux operating system and communicates with the PostgreSQL server by the standard JDBC protocol. The ArrayPlex client can be operated on any Mac OS X, Windows, or Linux computer. The ArrayPlex client is not installed but rather launched through use of Java Web Start, ensuring that the client is always up-to-date when used on any computer. The ArrayPlex client communicates with the ArrayPlex server by HTTP.

The ArrayPlex server is designed to operate on either the Linux operating system or Mac OS X (Figure 1) [7]. ArrayPlex includes Apache Tomcat [8] as the embedded application server, which awaits connections and responds to client data requests. The ArrayPlex server stores the majority of its managed data in the PostgreSQL relational database system [9].

The ArrayPlex client is a graphical user interface that contains dozens of data management, analysis, and visualization features. It is compatible with Mac OS X, Windows XP, Windows Vista and most distributions of Linux operating systems. It communicates by standard network protocols with the ArrayPlex server and, thus, can operate on any computer with network connectivity to the ArrayPlex server. Because it communicates with the ArrayPlex server using the same protocol a web browser utilizes, the ArrayPlex client requires no special changes to client firewall configurations or network settings for operation. The ArrayPlex client requires no local installation. The application resides on the ArrayPlex server and is remotely retrieved and launched through use of Java Web Start [10]. This ensures that with each execution the end-user is using the latest version of the ArrayPlex client. This design and implementation allows a large user group to share a customizable and expanding graphical user interface without the constant need for distributed upgrades or reinstallations with each cycle of improvement. In addition to the graphical user interface, ArrayPlex has a set of command-line executed client-side modules packaged in the form of standard Java Archive format (JAR) files [11]. These modules contain documented analytical routines that communicate with the ArrayPlex server exactly like the ArrayPlex client. This feature allows the distributed network design of ArrayPlex to be used by command-line application and script-driven analysis just as easily as the graphical interface.

### Bundled genomic resources

**Figure 2:**
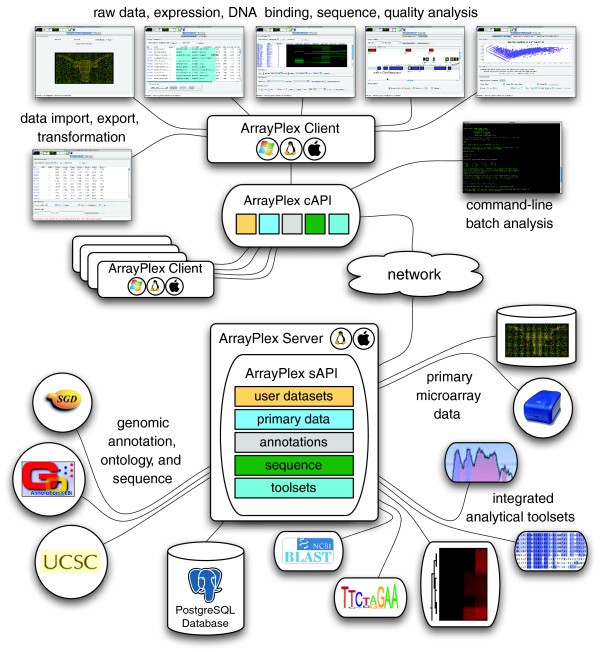
Architecture, resources, network-centric communication. The complete ArrayPlex environment is composed of the combination of the ArrayPlex application server and the many genomic resources and analytical toolsets that it installs, manages, and provides. The ArrayPlex server installs genomic annotations, ontological assignments, and genome sequence for supported organisms. Additionally, toolsets providing genomic sequence extraction, BLAST, sequence search, sequence discovery, and multi-sequence alignment are provided. Both the ArrayPlex client and command-line modules network-access these genome resources and analytical toolsets through the documented ArrayPlex API.

The complete ArrayPlex server meta-environment is composed of the ArrayPlex application server and many bundled genomic resources and analytical toolsets (Figure 2, Tables 1 and 2). The process of ArrayPlex server installation acquires each of the genomic resources (Table 1) from its officially hosted location. This includes generic Gene Ontology (GO) descriptors, organism-specific GO assignments, and organism-specific gene annotations.

All resources are processed from their heterogeneous downloaded forms to a structured query language (SQL) format that is loaded into the ArrayPlex relational database schema. The transformation removes all of the organism-specific nature of the data and allows the ArrayPlex programmatic API to be designed such that reusable code modules can be implemented independent of the original source of the annotations. A functional example of this would be GO assignments. This information is species-specific and details the mapping of universal GO terms to specific genes in a given organism. The downloaded forms of these assignments for human and mouse differ from yeast in format and content, because these assignments are curated and managed by independent research institutions: European Bioinformatics Institute for human and mouse, Stanford Genome Database for yeast. The transformation of this information to a single format and storage in a relational schema enabled a single set of ArrayPlex database source-code to be written to retrieve and use this information. This allows programmers using the ArrayPlex programmatic API to write data retrieval and analysis routines that are independent of the organism-specific caveats and institution-specific file formats. File format changes will be handled through alteration of the ArrayPlex parsing routines and released upgrades. These internal adaptations will be transparent to programmers using the API, thus shielding them from future file format evolution.

In addition to GO and gene annotations, complete genome sequence is downloaded for each of the supported model organisms. This genome sequence is in FASTA format but is converted to National Center for Biotechnology Information (NCBI) BLAST-database format by the ArrayPlex installation program using NCBI-provided utilities [12]. This transformation provides two advantages. First, it allows the ArrayPlex programmatic API to include complete BLAST functionality as a part of its catalogue of analytical operations. Second, and more importantly, it allows the ArrayPlex environment to take advantage of all the pre-existing NCBI-bundled toolsets for genome sequence retrieval.

Genome resources are most valuable when synchronized with the most recent versions available. Frequent modifications and additions occur to GO and other gene annotation assignments as they are continually curated and updated. In order to keep analysis routines and the resulting biological interpretations up to date, ArrayPlex is designed to not only download and store annotations upon system installation, but also to check for updated information, retrieve it, and update the resources managed within the relational schema. This functionality is provided and documented in the format of a standard system scheduler that is a part of the server operating system.

### Integrated open-source sequence analysis toolsets

In addition to the many genome resources hosted on the ArrayPlex server, a large number of open-source analytical toolsets are integrated into the environment (Table 2). This set of tools includes NCBI BLAST, cluster, CLUSTALW, AVID/rVista, and several sequence motif discovery applications: AlignAce, MDSCAN, and MEME [13-20]. As detailed in Table 2, the majority of these applications are downloaded, compiled from source-code, and installed by the ArrayPlex installation program. Licensing restrictions prevented this for a few of the integrated toolsets. Complete documentation is included with the ArrayPlex installation on how to retrieve and install these additional utilities. The inclusion of these toolsets transformed ArrayPlex from solely an information warehouse to a server capable of extended analytical capacity. All of these analytical features are accessible by way of the graphical ArrayPlex client application, the command-line modules, and the programmatic API. Such access facilitates centralized and coordinated high-throughput data and sequence operations such as sequence retrieval, data manipulation and transformation, multi-genome BLAST, sequence motif search and discovery, hierarchical clustering, and sequence alignment. For example, it is possible to retrieve genomic sequence upstream of a set of genes of interest and carry out sequence motif discovery, all based on a few user-defined parameters. All of these utilities are executed on the ArrayPlex server, with only the results being transmitted immediately to the client computer. Thus, client computers that might not be able to compile or run these large-scale functional analysis programs can still access all their power in real time, and programmatically if so desired.

### Analytical accessibility and customization

In addition to the many genome resources and toolsets hosted by the ArrayPlex environment, Figure 2 depicts the overall interactivity and relationship of the subcomponent elements. Both the ArrayPlex client and the command-line modules communicate over a network connection with the ArrayPlex server using the hypertext transfer protocol (HTTP). Many individual clients and/or command-line modules can simultaneously interact with a single server. On several occasions we have executed more than a dozen command-line modules simultaneously interacting with a single ArrayPlex server for annotation, ontology, and genome sequence, as well as analytical toolset executions. The ArrayPlex server was easily able to manage these parallel requests, some of which took days to weeks to complete.

**Figure 3:**
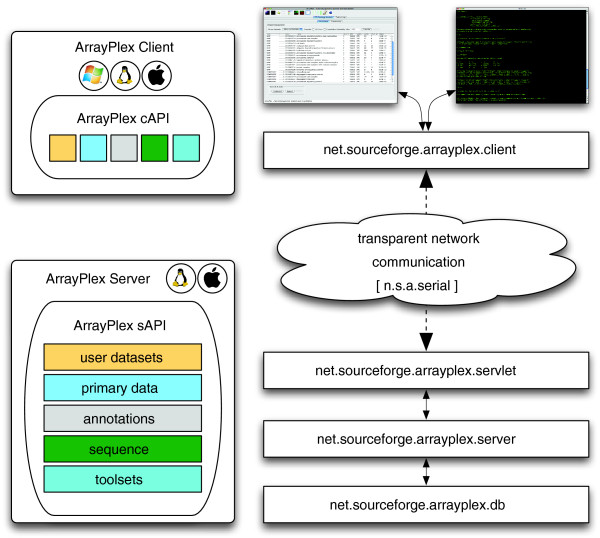
Matching graphical client and command-line utilities use the same API for communication with the server. The ArrayPlex client and command-line modules use the network capabilities of the ArrayPlex API to send requests and retrieve results.

Some client-side utilities such as sequence motif analysis are replicated between the graphical ArrayPlex client program and the command line modules. The former is useful for interactive and visual analysis while the latter facilitates flexible, programmatic execution. The ArrayPlex programmatic API mediates communication between both the client and the command-line module with the server (Figure 3). Each of these components interacts with the API by way of the [net.sourceforge.arrayplex.client] package of routines. These client routines are designed to marshal the input parameters, data, and named operations being sent to them in such a way that the ArrayPlex server can decode this information and respond. The objects exchanged between the client and server are an extensive and specialized set that is part of the [net.sourceforge.arrayplex.serial] package of resources. The [net.sourceforge.arrayplex.servlet] package receives requests and decodes both what part of the client API made the request and what specific information is being sent to facilitate its execution. The servlet API then calls a mirror server API, packaged as [net.sourceforge.arrayplex.server], where actual functional operations occur. This package contains dozens of classes that interact with the ArrayPlex server operating system to execute analytical tasks or with the ArrayPlex relational database API [net.sourceforge.arrayplex.db] to retrieve either user datasets or genomic annotations. When an analytical process completes or when information is retrieved, the process begins to fold back upon itself. Information is again loaded into API-based objects that are returned across the network to the original client operation.

This design and capacity is notable in two ways. First, the user invoking the client API routines needs no actual knowledge that the programmatic request will be fulfilled over a network on a remote server. The API is designed such that the complication of network implementation is hidden from the user. For example, the operation *executeBlastAll (organism, evalue, sequence)*, which is part of the *SequenceResources *client API, does not reveal to the programmatic user that, during its execution, the parameters *organism*, *evalue*, and *sequence *are encoded into an object and sent across the network to the ArrayPlex server where the NCBI-BLAST utility *blastall *is actually executed. The result of that *blastall *execution is then formatted into a programmatic object on the server, and returned across the network to the client computer. To the programmatic user of the client API no network operation is evident; the *BlastResult *object is the result of the operation and their programmatic routines move to the next step just as if everything executed and completed on their local computer. Second, the information that is exchanged with the ArrayPlex server is in the form of documented API objects. This increases the efficiency by which a programmatic user can utilize the ArrayPlex API compared to other methods that launch processes remotely and retrieve results locally. Most methods of remote task invocation require the user to parse a stream of resulting information that is returned from the server. The task of parsing this information and determining actual results is error-prone. The ArrayPlex APIs are designed to communicate in terms of API documented objects. In the example above, the *BlastResult *object that is returned from the ArrayPlex server is a programmatic object just like any other in the application environment. Referring to the provided documentation the programmatic user can find out that the *BlastResult *object is composed of a set of *BlastHit *objects, each of which has parameters describing the genomic loci where BLAST found matching sequences.

The entire ArrayPlex environment is designed to allow customization. The ArrayPlex client can incorporate internationalization and localization of language elements through modification of a single resource bundle containing nearly all labels that appear throughout its interactive graphical interface. Sections of the ArrayPlex client can be removed; newly designed sections can be accommodated.

### Documentation and guidance

**Figure 4:**
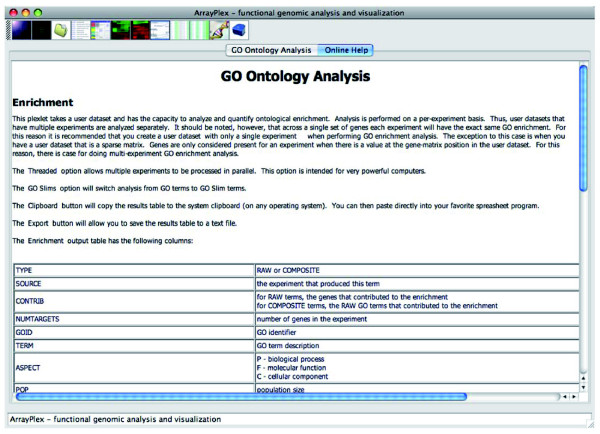
Multi-source documentation. All execution contexts within the ArrayPlex environment are documented. In this example, *Go Ontology Analysis *is documented from within the context of the ArrayPlex client (top). The bottom panel shows JavaDoc documentation of the API.

The analytical routines available in both the graphical client and command-line modules are documented. Execution of any of the command-line modules without arguments displays usage documentation. Similarly, the ArrayPlex client has hypertext-formatted help content for each of the interactive sections of the application. This content describes the analytical effect of chosen options and the meaning of results that are displayed (Figure 4). The programmatic API is similarly documented, detailing the parameters required by each API and the format and meaning of returned objects.

## Results and discussion

### Analytical proving ground

We have tested the entire ArrayPlex system - server resources, client, and all command-line modules - over the course of more than a year in a real-world research context. We recently described the reconstruction of a genome-wide transcriptional regulatory network based on integrating data from more than 600 individual microarray experiments covering more than 260 transcription factors [21]. ArrayPlex was the central hub of all computational activities for this project throughout each phase of data transformation and analysis.

We systematically screened hundreds of independent microarray experiments for channel-specific signal bias. We used a sophisticated error model implementation to identify statistically significant target genes based on replicate microarray data. With target genes identified for each of the 260 transcription factors profiled, we carried out regulatory epistasis analysis, expansive GO enrichment analysis, characterized sequence motif search, and novel sequence motif discovery. Additionally, ArrayPlex format-conversion capabilities were used to elucidate significant novel transcription factor-to-factor regulatory insights.

The ArrayPlex command-line modules *ErrorModel.jar*, *InteractionGraph.jar*, and *TargetAnalysis.jar *(Table 3) were developed concomitant to ArrayPlex and were employed for all the operations that led to the resulting biological conclusions. These modules are included as part of the ArrayPlex set of command-line functions as their capacity is useful for most gene expression analysis. Additionally, the command-line modules *AnnotationResources.jar*, *DatasetOperations.jar*, and *SequenceAnalysis.jar *provide application-neutral implementation methods to expose the genomic resources and open-source toolsets hosted by the ArrayPlex server to the command-line module user.

**Table 3:** Command-line modules

Module name	Purpose	Class
*AnnotationResources.jar*	Genome annotation and ontology retrieval	Generic
*DatasetOperations.jar*	User dataset retrieval, transformation, and manipulation	Generic
*SequenceAnalysis.jar*	Genome sequence extraction, search, discovery, manipulation	Generic
*ErrorModel.jar*	Example routines in replicate combination	Regulation
*InteractionGraph.jar*	Example routines in network modelling	Regulation
*TargetAnalysis.jar*	Example routines in ontological and sequence analysis	Regulation

The six command-line modules built by and provided with the ArrayPlex installation. The first three modules, classified as 'Generic', are most useful for command-line access to any of the resources hosted on the ArrayPlex server. This includes all genome sequence, annotation, ontology, and user dataset information. The *SequenceAnalysis.jar *module additionally contains all of the genome sequence operations featured in the ArrayPlex client, including organism-specific sequence extraction, BLAST, known-motif search, *de novo *motif discovery, and multi-sequence alignment. The modules classified as 'Regulation' are useful for analysis of regulator-target relationships as illustrated in our recent reconstruction of a functional transcriptional regulatory network [21]. They provide reusable analytical operations and illustrate how the ArrayPlex programmatic API can be used for constructing novel analysis routines.

### High-throughput microarray data quality analysis

One important step in most DNA microarray analysis is that of data quality evaluation. For example, it is important to check for any signal intensity bias and understand the effect of data normalization on individual and entire batches of microarray experiments. Secondarily, the selection of significant microarray values for an individual or set of experiments involves the filtering of candidate spots based on a variety of spot metrics. Measurements such as signal to noise ratios, spot consistency regression correlations, and background subtracted single-channel intensity values are typical metrics that are used to separate statistically meaningful spot values from those of dubious quality.

To address these issues we developed an entire section of the ArrayPlex client dedicated to processing, statistical analysis, and visualization of large batches of input data. The *GenePix Results File Operations *section of the ArrayPlex client has the capacity to batch-process a large number of *GenePix Results *(GPR) files for quality control evaluation. First, the *GenePix Results File Charting *section can read sets of GPR files into a batch queue for graphical analysis, such as generating MA plots (spot fluorescent intensity A to log-ratio M), which can detect a bias in the relationship of absolute signal intensity to ratio of spots [22]. In addition to MA plots, histograms and scatter-plots can be mass-produced for any of the dozens of GPR spot metrics, enabling detection of biased signal-to-ratio relationships, non-normal log-ratio distributions, and substandard signal to noise distributions with the selection of just a few parameters and the browsing of automatically saved images.

**Figure 5:**
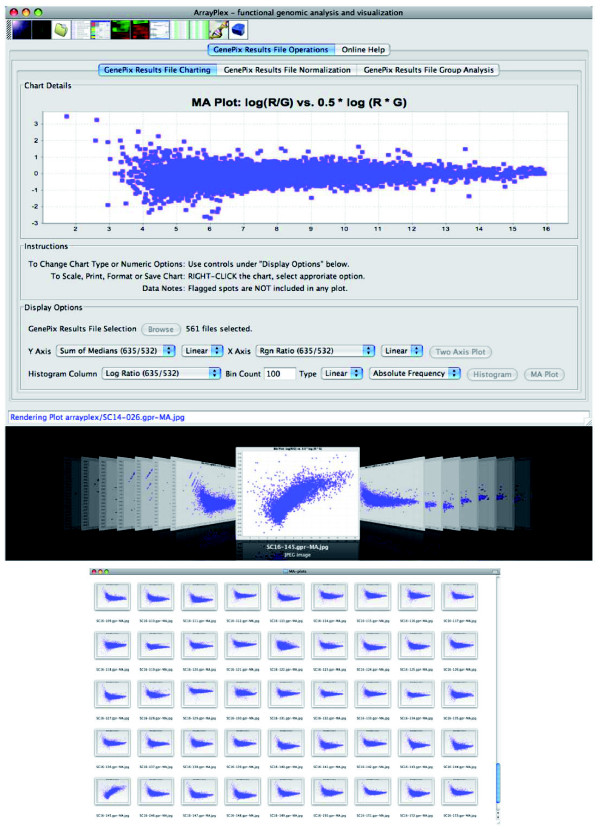
Result file batch quality visualization. The *GenePix Results File Charting *section of the ArrayPlex client contains extensive resources for the statistical and visual processing of *GenePix Results *files (GPR). Batch production of quantitative visualizations such as MA plots, scatter-plots, and spot-metric histograms are possible. All graphs can be exported as JPG-formatted images in batch mode to a given folder and browsed using the standard thumbnail capability of the client operating system. This provides the capacity to screen for a number of data quality attributes in large sets of DNA microarray experiments.

Each of the more than 600 individual microarray experiments were screened for channel-specific signal bias and a variety of other possible data irregularities using the high-throughput batch functions provided by the *GenePix Results File Charting *section of the ArrayPlex client (Figure 5). MA plots were generated *en masse *and used to screen for intensity-dependent spot-ratio biases while log-ratio histograms provided the ability to visually detect unexpected ratio distributions. Individual experiments with obvious bias were eliminated from the process of replicate combination and significant target determination.

**Figure 6:**
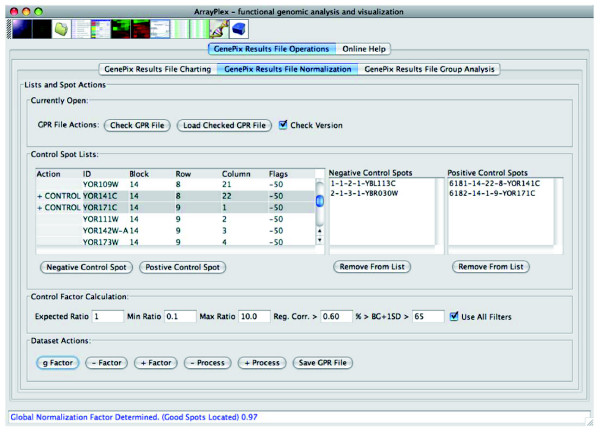
Result file normalization. The *GenePix Results File Normalization *section of the ArrayPlex client provides varied resources for the normalization of *GenePix Results *files (GPR). Positive control, negative control, and global median normalization options are available. Configurable statistical filters provide the capacity to threshold spot quality in the selection of microarray spots used to determine normalization coefficients. Export of normalized datasets is in the same format as that of the input data (GPR). This allows ArrayPlex to be part of a flexible pipeline of data analysis and processing.

The *GenePix Results File Normalization *section of the ArrayPlex client has the capacity to read, normalize, and save processed results in *GenePix Results File *(GPR) format through the implementation of three selectable algorithms: positive control, negative control, and global mean distribution adjustment. The functions of this section of the ArrayPlex client provide a novel capacity not present in any software package or microarray database. Determination of normalization coefficients and subsequent data adjustment is based upon interactive and controllable selection of positive and negative control microarray spots as well as user-selectable spot-quality metrics. A researcher is not limited to the blind dictation of parameters by which normalization coefficients will be imposed on primary data, but rather has the capacity to interactively explore the effects of these parameters and then decide which values are appropriate (Figure 6). Once filtering metrics have been determined, the process of normalization and results export remains in the native GPR format of the original input data. The ArrayPlex client thus serves as a normalization intermediary without interfering in the process of storing final results in one of many possible microarray databases that supports the GPR file-format.

**Figure 7:**
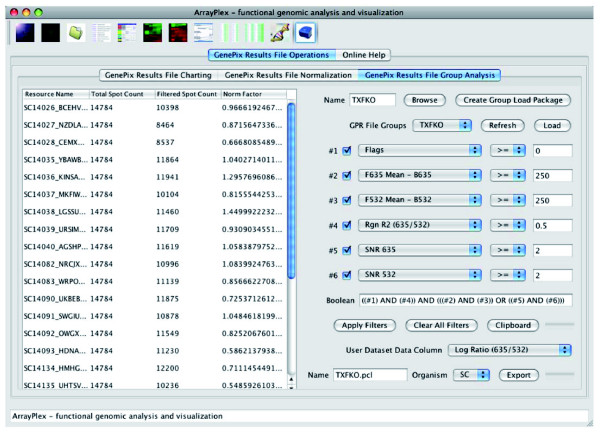
Result file aggregation, filtering, and data extraction. The *GenePix Results File Group Analysis *section of the ArrayPlex client allows for the aggregation of individual microarray datasets (*GenePix Results *files) into named and dynamically loadable file groups. A loaded file group can be interactively filtered through logical combination of spot-metric filters. Filter toggling can be used to dynamically determine the relative contribution of individual filters on the sum total of spots excluded for each microarray experiment in a file group. Once appropriate filters have been determined, datasets are matrix-exportable for any of the dozens of *GenePix Result File *spot metrics.

Interactive exploration of filter-mediated spot-exclusion and tabular data export across grouped primary datasets is a powerful feature found in the *GenePix Results File Group Analysis *section of the ArrayPlex client not present in open-source or commercial software counterparts. Primary datasets in the form of an unrestricted number of GPR files are aggregated, named, and permanently stored in the ArrayPlex server-managed relational database as a *GenePix Results File Group*. The file group, once stored, is available for dynamic loading into the ArrayPlex client at any time (Figure 7). The loading of a file group is the first step in filtered tabular export of a chosen *GenePix *spot-metric across all experiments contained within the group. The impact of statistical filtering as it relates to spot exclusion is interactively adjustable through a set of user-controlled and logically configurable primary data filters. A researcher has the capacity to define and combine filters and receive immediate feedback regarding what proportion of each dataset within the file-group the chosen filter thresholds would exclude. Thresholds can thus be carefully studied and chosen in a way that provides unprecedented transparency to the process of primary data filtering. After appropriate filters and thresholds have been determined and applied, the resulting data matrix is exported in a standard tabular PCL (pre-clustering) file-format.

The feature-set provided by the *GenePix Results File Group Analysis *section of the ArrayPlex client was invaluable in the earliest stages of transcription factor knock-out primary data aggregation and processing. Implementation of the error model required the systematic construction of several separate primary data matrices for the hundreds of individual microarray experiments that were the input to this stage of data processing. These included channel-specific foreground intensity, background intensity, and signal-to-noise matrices as well as spot-quality metrics such as regression correlation. ArrayPlex allowed us to explore and aggregate hundreds of individual microarray experiments as a single unit through importation as a single file group. Once the file group was created we were able to study the dataset-specific effects of various spot-metric thresholds on matrix construction and filter-mediated spot exclusion. These features impacted our understanding of both individual experiments as well as sets of microarray hybridizations performed together as batch groups. For each of the candidate statistical thresholds that were under consideration, we were able to understand the proportion of spots that would be excluded from individual experiments as well as gain visibility as to which batch groups were the most susceptible to filter-induced data exclusion. Filter toggling allowed us to clearly understand which individual filters in a logical group were having the most impact on spot exclusion. Once we arrived at a set of thresholds we deemed functionally appropriate, we then exported internally consistent data-matrices for each of the spot-metrics required by the error model. This section of the ArrayPlex client was so effective for these operations that it replaced our microarray database (Longhorn Array Database) for all data aggregation, filtering, and filtered dataset extraction portions of this research initiative.

### Ontological enrichment and connectivity

A successful component of the reconstruction of the functional regulatory network was the mining of GO assignments among the target genes of a given transcription factor for statistically significant GO term enrichment [21]. This functionality is built into both the ArrayPlex client and the command-line module *TargetAnalysis.jar*. The command-line module *AnnotationResources.jar *has the supplemental capacity to return a normalized single-format set of both ontology term declarations and organism-specific term assignments for each of the supported organisms.

The high-throughput capacity of the GO term enrichment toolsets provided by the command-line module *TargetAnalysis.jar *allowed us to calculate statistical enrichment for regulated target sets of each of the hundreds of transcription factors characterized. The process was simplified and easily repeatable through the module-provided ability to process input as a single file for all transcription factor target sets. Execution time was significantly reduced through parallel multi-threaded processing functionality provided as a user-selectable option. Configurable ArrayPlex server-mediated maintenance of GO terms and assignments ensured that up-to-date information was provided for each repetition of the analytical workflow.

The GO term enrichment toolsets contained the novel capacity to evaluate both individual (RAW) and network-aggregate (COMPOSITE) terms for statistical enrichment. The capacity for GO term enrichment calculations to evaluate both individual and aggregate terms ensured that ontology terms that were proximally co-located in the GO hierarchy were mined for statistical significance. Individual terms that might have missed statistical thresholds for significance were evaluated for the ability to network-aggregate up to significant higher-order terms.

**Figure 8:**
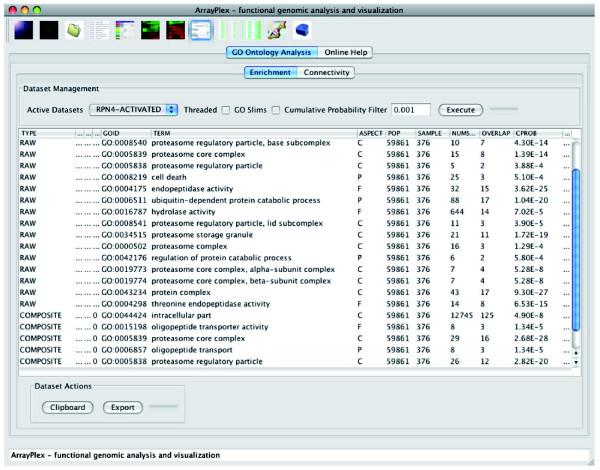
GO ontological assignment enrichment, connectivity. The ArrayPlex client performing GO term enrichment for the transcription factor RPN4. Specific individual (RAW) and network-aggregate (COMPOSITE) GO terms relating to the characterized roles in proteasome biogenesis, ubiquitination, and catabolic protein degradation are recovered and statistically enriched as evaluated by the cumulative hypergeometric probability.

The ArrayPlex client processing the set of primary regulated targets for the transcription factor *RPN4 *is depicted in Figure 8. The cumulative hypergeometric probability was used to evaluate both individual and network-aggregate terms for statistical enrichment. As depicted, the set of *RPN4 *primary targets were significantly enriched for many terms related to the proteasome, ubiquitination, and catabolic protein degradation. This characterization of *RPN4 *correlated well with its previously established biological role in proteasome biogenesis and protein degradation [23]. In total, 156 of the more than 200 transcription factors profiled showed statistical enrichment for 213 GO terms at a Bonferroni-corrected *P*-value threshold of 4.0 × 10^-5^[21].

### Sequence analysis - discovery and search

We used ArrayPlex exclusively in the analysis of promoter sequences of regulated targets for each profiled transcription factor. We wished to determine whether sets of regulated targets had novel over-represented sequence motifs in their respective promoter regions. Similarly, we used ArrayPlex to characterize the statistical over-representation of previously characterized motifs within these regulatory regions. The module *TargetAnalysis.jar *provides the transcription factor target-specific implementations of these methods developed during this study while more generic implementations of these services are available in the module *SequenceAnalysis.jar*.

Sequence sets for each regulated target pool were extracted using ArrayPlex genome sequence retrieval services - available through the ArrayPlex client, command-line modules, and programmatic API. These sequence sets were then re-submitted to the ArrayPlex server for *de novo *analysis using APIs that invoked the bundled toolsets AlignACE, MEME, and MDscan. Each of these programs is a motif-discovery application designed to use a background sequence model to find over-represented motifs within the set of sequences provided. The background sequence model we utilized was a nucleotide frequency matrix computed by extraction of all *S. cerevisiae *intergenic regions. Each was permuted by a set of module-accessible parameters, including the desired motif width and the number of expected motifs. The output from this process was converted by ArrayPlex from the native output of each of the motif-discovery toolsets to a single universal format, thereby significantly simplifying downstream analysis. Ultimately, 105 transcription factor target sets produced 490 statistically significant and novel sequence motifs that passed systematic evaluation over 400,000 candidates sequences for properties such as nucleotide complexity and motif length [21].

A motif search process was executed for each transcription factor target set to scan promoter regions for the existence of previously characterized *cis*-regulatory sequences. A set of consensus sequences for each of the transcription factor deletions was aggregated from Stanford Genome Database, The Promoter Database of *Saccharomyces cerevisiae *(SCPD), and previous research [24-27]. Each of the consensus sequences was used by ArrayPlex to synthesize a regular expression that was evaluated for statistical enrichment relative to the previously described background model. Of the more than 200 profiled, 102 transcription factor target-set promoter regions showed enrichment for previously characterized sequence motifs, thereby increasing confidence in both the biological validity of the characterized motifs and the target pools characterized by this study [21].

Each of these sequence analysis processes was made possible and iteratively repeatable through the on-demand and up-to-date genome sequence resources offered by the ArrayPlex server, the parametric options available in its command-line modules and programmatic API, and the bundled sequence discovery and search toolsets.

### Visualization - regulator on regulator analysis

**Figure 9:**
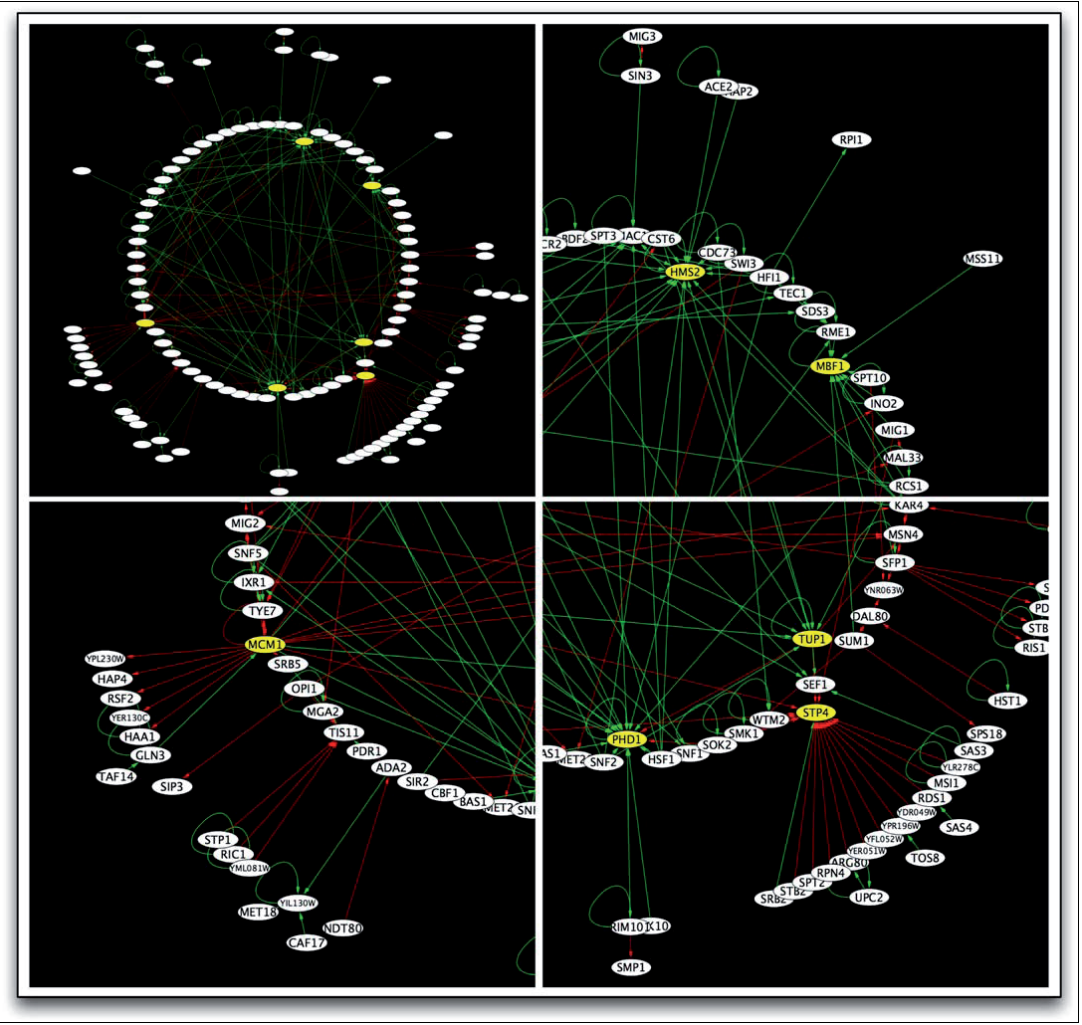
Regulator on regulator visualization. The command-line module *InteractionGraph.jar *has the capacity to convert to visualization-ready file formats that can be read by Cytoscape for visualization [28]. The functional transcriptional regulatory network dataset was filtered to show regulatory interactions only among transcription factors [21]. Red connections indicate activation while green are indicative of repression. The arrowhead points from the regulator to the target that is being regulated. Certain transcription factors are predominantly regulators of other transcription factors or regulated by other transcription factors.

The command-line module *InteractionGraph.jar *has the capacity to cross-convert between many commonly used primary data formats, such as the PCL format common to many DNA microarray analysis applications and the graph-markup language format (GML) common to many network-visualization packages such as Cytoscape [28]. In order to identify sub-network relationships where transcription factors regulate one another, we recently filtered the large-scale transcription factor deletion data so as to include only gene targets that were themselves transcription factors. In this manner, the filtered datasets were limited to represent the inter-regulatory interactions of transcription factors. We were interested in whether certain transcription factors acted as network hubs, concentrated points of in-bound or out-bound transcriptional activation or repression. In order to visualize the filtered network relationships, we converted the raw PCL to a GML format and used Cytoscape to render the resulting network (Figure 9).

The transcription factors PHD1, STP4, MCM1, MBF1, and HMS2 each have either a significant count of in-bound or out-bound regulatory connections with the other transcription factors that were profiled [21]. Specifically, MCM1 activates a large number of transcription factors while STP4 is conversely activated by a large number of transcription factors. It is not surprising that MCM1 appears to be an activation hub for many transcription factors in the larger regulatory network. MCM1 has been shown to perform an active role in cell-cycle regulation through regulation of DNA replication initiation [29]. STP4 has little official annotation. GO term enrichment analysis of its affected targets indicates statistically significant roles in nucleotidyltransferase, polyamine transporter, spermine transporter, and polyamine activities. These activities are general to the many pathways of amino acid metabolism and it is thus not surprising that STP4 would then be activated by a wide variety of other transcription factors. Also of interest in the regulatory network, the transcription factors MBF1, PHD1 and HMS2 are each repressed by many factors. Both PHD1 and HMS2 have been shown to perform an active role in pseudohyphal growth adaptation [30-32]. It is reasonable to believe that their transcriptional abundance would be repressed by many transcription factors in the many conditions in which their cellular role is not required.

It would have been difficult to detect these nuanced relationships without this rendering. The capacity for ArrayPlex to interconvert file formats from primary data formats to visualization-ready formats increases the efficiency and flexibility of data exploration and analysis.

### Comparison to similar software packages

While ArrayPlex provides features common to many commonly utilized microarray databases (Bioarray Software Environment, Stanford Microarray Database, Longhorn Array Database), the ArrayPlex environment is not intended to operate as one. ArrayPlex was developed to fulfil the need for interactive, command-line, and programmatic access to up-to-date genomic resources and analytical toolsets in a networked computational environment. Thus, while several ArrayPlex client functions such as hierarchical clustering, ontology analysis, and *GenePix Result File Group Analysis *have the intrinsic capacity to store proprietary and tabular microarray data, ArrayPlex was not designed to supplant functionality nor provide this capacity in a manner that is comparable to traditional microarray databases. Several other research projects address some of these goals in a variety of ways, but none provide the combined suite of resources that ArrayPlex does. EnsMart, Atlas, Mayday, SeqHound, EMMA, GEPAS, and DAVID are all examples of bioinformatic server environments that address many of the stated data association and analytical goals [33-39]. SeqHound and Atlas each house an extensive API-accessible list of resources yet lack both an extensible user interface and pre-defined command-line modules. EnsMart, EMMA, and GEPAS each have a web interface or command-shell environment but lack a client-server enabled API. This feature was core to ArrayPlex's design goal of enabling all computers in a research environment to be productive platforms on which data analysis can be accomplished. Mayday and DAVID are toolsets focused upon DNA microarray data analysis and GO analysis, respectively. They each are feature-rich in these categories but lack integration with the wide variety of genomic resources provided by the ArrayPlex environment.

The high-throughput quality evaluation capabilities of the *GenePix Results File Operations *section of the ArrayPlex client, command-line modules, and programmatic API surpass existing commercial and open-source software offerings and greatly reduce the time and error involved in screening large microarray datasets for signal bias. Molecular Devices, the original manufacturer of *GenePix Scanners*, provides similar quality evaluation features in its *GenePix Pro *and *Acuity *software packages. These features, however, are limited to graphical user interface access and low-throughput single-microarray analysis. Bioconductor is an open-source microarray data analysis environment that offers programmatic API access to software routines capable of high-throughput quality evaluation and plot generation similar to that of ArrayPlex [40]. Use of Bioconductor, however, is not extended to a graphical user interface or a simplified command-line module. In this manner, Bioconductor requires specialized knowledge of both the R programming language and shell environments, and is thus an option suited primarily for experienced computational biologists and programmers. Finally, in addition to quality evaluation, the *GenePix Results File Normalization *and *GenePix Results File Group Analysis *modules of the ArrayPlex client provide varied normalization, filter-based evaluation, and extraction features only partially provided by commercial software packages.

## Conclusion

The ArrayPlex environment is a robust platform for genomic data analysis and visualization. Its ease of installation and operation provide ready-to-use aggregated genome resources, genome sequence, and analytical toolsets to users of the graphical interactive ArrayPlex client, command-line modules, and programmatic API. The ArrayPlex server keeps managed genome resources up-to-date, thus providing information and analytical results that are synchronized with curated knowledge. The open-source programmatic API allows all of the ArrayPlex functions, both client and server, to be expanded. ArrayPlex has been tested and improved in the course of a large-scale research project involving the utilization of all of its genome resources, genome sequence, and analytical toolsets.

## Requirements and availability

ArrayPlex is available from its project site at sourceforge.net [41]. The ArrayPlex server, client, and command-line modules are included in a single installation package. The ArrayPlex client and the command-line modules are prepared during the process of ArrayPlex server installation such that they are configured to communicate with the ArrayPlex server being installed by the system administrator. Complete source-code is provided for each of the operational components.

### ArrayPlex server requirements

The default server installation requires either an Intel-based computer running the Linux operating system or any computer running Mac OS X. Linux servers running both the 2.4 and 2.6 generation of kernels have been tested. During its development, ArrayPlex was operated on many distributions of Linux [42-46]. Mac OS X has been tested with version 10.4 (Tiger), but we expect that most generations of this operating system will be compatible. Additionally, an operational PostgreSQL relational database system is required. The ArrayPlex development and testing process has utilized PostgreSQL server versions from 7.3 to 8.2. The database server does not need to be installed on the same computer as the ArrayPlex server, only reachable by TCP/IP network connectivity and standard PostgreSQL client utilities. A sequestered ArrayPlex schema instance is created within the PostgreSQL database server such that ArrayPlex can co-exist with other database instances. Neither the Java Runtime Environment (JRE) nor Apache Tomcat needs to be separately installed by the user, since each of these resources is bundled within the ArrayPlex installation in order to create a more encapsulated and ready-to-operate system. Alternative implementations of the JRE or Apache Tomcat can be substituted through simple sub-folder replacement within the installed ArrayPlex server. This process is documented in the *ArrayPlex Server Installation Guide*.

The ArrayPlex distribution, as downloaded from the SourceForge.net project site, is 350 MB in size. The ArrayPlex server, however, downloads a large quantity of genomic annotation and sequence during the installation process. The genomic sequence files are transformed into NCBI BLAST-compatible databases that allow for rapid sequence retrieval. This results in the consumption of significant drive space such that an operational ArrayPlex server requires at least 14 GB for complete installation.

### ArrayPlex client requirements

The ArrayPlex client is not installed but rather launched from the ArrayPlex server by clicking a link within any web browser. The client is supported on Mac OS X, Windows XP, Windows Vista, and most distributions of the Linux operating system. Each of these client operating systems must have a JRE installed [47]. The default Microsoft-provided Java installation on any version of Windows is not supported. A JRE should be downloaded and installed from Sun Microsystems. The JRE that is bundled with Mac OS X (10.2 Jaguar to 10.5 Leopard) has been tested.

### Command-line module requirements

The requirements for use of the command-line modules match those of the ArrayPlex client. They are built by the ArrayPlex server installation process and downloaded by a web-browser to any supported client computer.

## Abbreviations

API: application programming interface; GML: graph-markup language; GO: Gene Ontology; GPR: *GenePix Results*; HTTP: hypertext transfer protocol; JRE: Java Runtime Environment; NCBI: National Center for Biotechnology Information; PCL: pre-clustering file format.

## Authors' contributions

PJK designed, implemented, and documented all components of the ArrayPlex environment. PJK and VRI wrote the manuscript. VRI provided overall supervision and funding of the project.
